# Three New Supramolecular Coordination Polymers Based on 1H-pyrazolo[3,4-b]pyridin-3-amine and 1,3-benzenedicarboxylate Derivatives

**DOI:** 10.3390/polym11050819

**Published:** 2019-05-07

**Authors:** Yun Xu, Qing-Hua Deng, Fang Ding, Ran An, Dong Liu, Ti-Fang Miao

**Affiliations:** College of Chemistry and Materials Science, Huaibei Normal University, Huaibei 235000, China; 13365613602@163.com (Q.-H.D.); pangao_1992@163.com (F.D.); raner28@163.com (R.A.); dongliu@chnu.edu.cn (D.L.); miaotifang@163.com (T.-F.M.)

**Keywords:** supramolecular coordination polymers, nonanuclear zinc unit, hydrogen-bonding interactions, photoluminescence

## Abstract

Three new supramolecular coordination polymers, namely [Zn(1,3-BDC)(HL)]*_n_* (Polymer 1), [Zn_3_(1,3,5-BTC)_2_(HL)_2_(H_2_O)_2_]*_n_* (Polymer 2), and [Zn_9_(5-SO_3_-1,3-BDC)_2_(L)_8_(OH)_4_]_n_ (Polymer 3), were synthesized under solvothermal conditions, based on 1H-pyrazolo[3,4-b]pyridin-3-amine (HL) along with 1,3-benzenedicarboxylate (1,3-BDC) and its derivatives, such as 1,3,5-benzenetricarboxylate (1,3,5-BTC) and 5-sulfo-1,3-benzenedicarboxylate (5-SO_3_-1,3-BDC). Polymers 1–3 were characterized by elemental analysis, IR spectroscopy, powder X-ray diffraction (PXRD), and single crystal X-ray diffraction analysis. Polymer 1 exhibited a two-dimensional (2D) 4-connected sql net. The neighboring 2D nets were further linked into a 3D supramolecular network by hydrogen-bonding interactions. Polymer 2 displayed a 3D (4, 4, 4)-connected network, which was further stabilized by $R_{2}^{2}$(14) and S(9) hydrogen-bonding rings along with π–π interactions. The 2D sheet structure of Polymer 3 was constructed by novel quasi-linear nonanuclear Zn(II) units, which further extended into a 3D supramolecular structure by hydrogen-bonding interactions. The solid-state photoluminescence properties of Polymers 1–3 were also investigated.

## 1. Introduction

Crystal engineering, based on coordination polymers, is one of the most popular research fields, because supramolecular coordination polymers possessing covalent and noncovalent interactions (such as hydrogen-bonding interactions and π···π-stacking interactions) have the advantages of both coordination polymers and supramolecular materials. Many of them not only have diverse and biomimetic dynamic structures but also have widely promising applications in many fields, such as in structural transformation (SCSC), ionic diodes, and semiconductors [1,2,3,4,5,6,7,8,9,10]. Although there are many factors affecting the structure of supramolecular coordination polymers, such as pH, temperature, and solvents, the most important one is the structure of ligands. Aromatic acid ligands and N-heterocyclic ligands are the best choice among many ligands, because carboxylic acid ligands and N-heterocyclic ligands have a variety of coordination models; furthermore, they can perform hydrogen bonding and aromatic π–π stacking in the construction of polymers, which are superior conditions for the synthesis of supramolecular coordination polymers [11,12,13].

**Figure 1:**
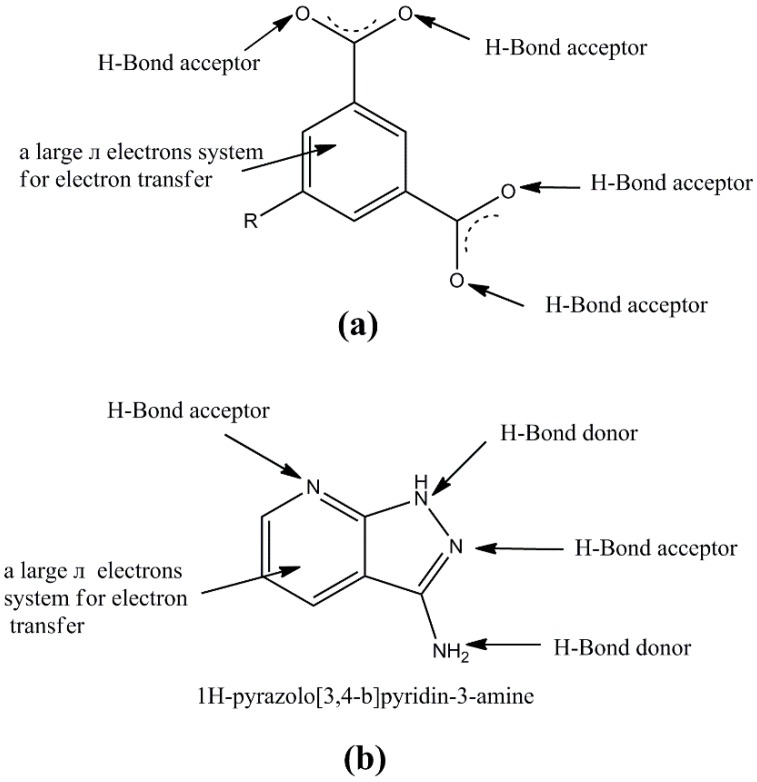
(**a**) Possible noncovalent interactions in ligands of 1,3-benzenedicarboxylate derivatives and (**b**) possible noncovalent interactions in the ligand of 1H-pyrazolo[3,4-b]pyridin-3-amine.

**Figure 2:**
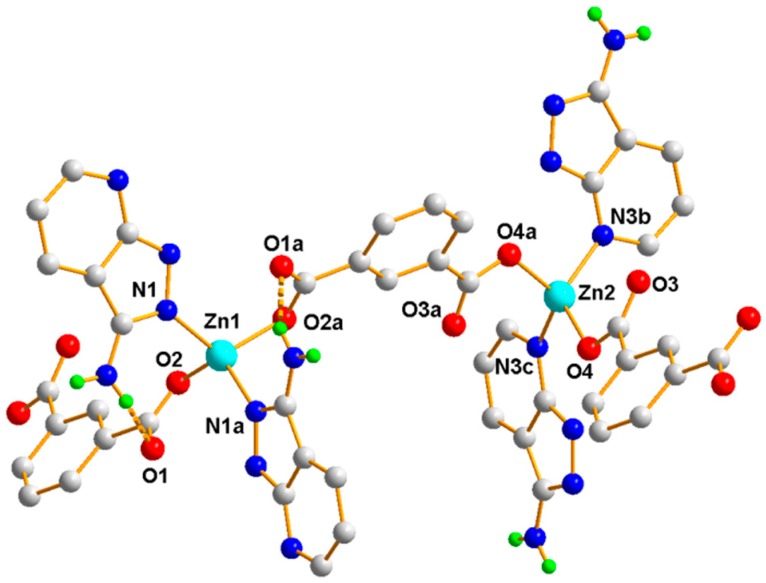
The coordination environment of the Zn(II) ions in Polymer 1. The brown dotted lines depict the hydrogen-bonding interactions, while other hydrogen atoms have been omitted for clarity.

Inspired by the information above, we selected 1,3-benzenedicarboxylate (1,3-BDC) and its derivatives—1,3,5-benzenetricarboxylate (1,3,5-BTC) and 5-sulfo-1,3-benzenedicarboxylate (5-SO_3_-1,3-BDC)—as multidentate aromatic carboxylic acids and the multidentate 1H-pyrazolo[3,4-b]pyridin-3-amine as a co-ligand to synthesize supramolecular coordination polymers. The main considerations were as follows: (1) these aromatic carboxylic acids not only have versatile bridging modes (monodentate, bidentate (*syn*−*syn*, *syn*−*anti*, and *anti*−*anti*), tridentate, and more) but can also form metal clusters and bridge structures to increase the rigidity and dimension of the main structure; furthermore, they can perform hydrogen-bonding interactions and π–π interactions with neighboring molecules [14,15,16], as depicted in Figure 1a, (2) 1H-pyrazolo[3,4-b]pyridin-3-amine ligand also has multidentate coordination sites [17,18], which can form various structures by coordination or hydrogen-bonding interactions, as depicted in Figure 1b; moreover, the pyrazole and pyridine rings in the HL ligands tend to form π–π interactions in assembling the coordination polymer, which may result in sensing properties [19], and (3) the rigidity and denticity of two kinds of ligands may contribute to some interesting properties.

On the other hand, d^10^-containing coordination polymers have obtained much attention due to their various coordination numbers, which can form a variety of coordination geometries, such as tetrahedral, trigonal bipyramidal, and octahedral. They are also desired for their prospective applications in biomedicine, luminescent materials, and nonlinear optics (NLO) materials [20,21].

To further understand the supramolecular coordination chemistry of rigidity in multidentate aromatic carboxylic acids and multidentate N-containing ligands in a reaction with Zn^2+^ ion, three new Zn(II) coordination polymers, formulated as [Zn(1,3-BDC)(HL)]_n_ (Polymer 1), [Zn_3_(1,3,5-BTC)_2_(HL)_2_(H_2_O)_2_]_n_ (Polymer 2), and [Zn_9_(5-SO_3_-1,3-BDC)_2_(L)_8_(OH)_4_]_n_ (Polymer 3), were successfully synthesized. The photoluminescence properties of the solid-state in Polymers 1–3 were also studied.

## 2. Experimental

### 2.1. Materials and Instruments

All reagents used for the synthesis (including metal salts (Meryer, Shanghai, China)), and organic ligands (J&K Chemicals, Beijing, China) were purchased commercially and used without further treatment. The IR spectra of Polymers 1–3 were measured on a Nicolet Magna-IR 560 Infrared spectrometer (Thermo Fisher Scientific, Waltham, MA, USA) at the range of 4000~400 cm^−1^. Powder X-ray diffraction measurements were carried out by using Cu Kα (λ = 1.54060 Å) radiation on a D/Max-2500 X-ray diffractometer (Rigaku, Tokyo, Japan). The elemental analyses (C, H, and N) were determined by an EA1110 CHNS elemental analyzer (CE instruments, Milan, Italy). Solid-state photoluminescence spectra were studied by using a Jasco FP-8600 fluorescence spectrophotometer at ambient temperature. Thermal analysis was performed on a Perkin-Elmer TGA-7 thermogravimetric analyzer (PerkinElmer, Waltham, MA, USA) with a heating rate of 10 °C/min.

### 2.2. Preparation

#### 2.2.1. Synthesis of [Zn(1,3-BDC)(HL)]_n_ (Polymer 1)

Yellow crystals of [Zn(1,3-BDC)(HL)]_n_ (Polymer 1) were obtained by dissolving Zn(NO_3_)_2_∙(H_2_O)_6_ (0.074 g, 0.25 mmol), 1,3-benzenedicarboxylic acid (0.042 g, 0.25 mmol), and 1H-pyrazolo[3,4-b]pyridin-3-amine (0.034 g, 0.25 mmol) in 10 mL H_2_O at 160 °C for three days. Crystals of Polymer 1 were filtered off and dried in air. Yield: 47% (based on Zn). Elemental analysis for C_14_H_10_N_4_O_4_Zn (%) Calcd.: C, 46.24; H, 2.77; and N, 15.41%. Found: C, 46.21; H, 2.79; and N, 15.39%. IR (KBr): υ(cm^−1^) = 3414(m), 1614(s), 1566(s), 1384(s), 1295(m), 1271(m), 1077(m), 954(m), 806(m), 744(m), and 718(m).

#### 2.2.2. Synthesis of [Zn_3_(1,3,5-BTC)_2_(HL)_2_(H_2_O)_2_]_n_ (Polymer 2)

Polymer 2 was synthesized in a method similar to that described for the preparation of Polymer 1, using Zn(NO_3_)_2_∙(H_2_O)_6_ (0.112 g, 0.375 mmol), 1,3,5-benzenetricarboxylic acid (0.053 g, 0.25 mmol), and 1H-pyrazolo[3,4-b]pyridin-3-amine (0.034 g, 0.25 mmol). Yield: 43% (based on Zn). Elemental analysis for C_30_H_22_N_8_O_14_Zn_3_ (%) Calcd.: C, 39.39; H, 2.42; and N, 12.25%. Found: C, 39.35; H, 2.45; and N, 12.22%. IR (KBr): υ(cm^−1^) = 3458(m), 3347(m), 3320(m), 1614(s), 1565(s), 1437(s), 1328(s), 1289(m), 1266(m), 1183(m),1107(m), 1076(m), 918(m), 799(m), 766(m), 730(m), 578(m), and 555(m).

#### 2.2.3. Synthesis of [Zn_9_(5-SO_3_-1,3-BDC)_2_(L)_8_(OH)_4_]_n_ (Polymer 3)

Polymer 3 was synthesized in a method similar to that described for the preparation of Polymer 1, using Zn(NO_3_)_2_∙(H_2_O)_6_ (0.112 g, 0.375 mmol), 5-sulfo-1,3-benzenedicarboxylic acid (0.062 g, 0.25 mmol), and 1H-pyrazolo[3,4-b]pyridin-3-amine (0.034 g, 0.25 mmol). Yield: 56% (based on Zn). Elemental analysis for C_64_H_50_N_32_O_18_S_2_Zn_9_ (%) Calcd.: C, 34.81; H, 2.28; and N, 20.30%. Found: C, 34.77; H, 2.31; and N, 20.26%. IR (KBr): υ(cm^−1^) = 3409(m), 3327(m), 1613(s), 1565(s), 1500(m), 1437(s), 1358(m), 1313(m), 1162(m), 1130(m), 1036(m), 951(m), 802(m), 765(m), 729(m), 368(m), and 619(m).

### 2.3. X-ray Crystallographic Determinations

Single crystal analyses for Polymers 1–3 were detected on a Bruker D8-QUEST diffractometer equipped with graphite monochromatic MoKa radiation (λ = 0.71073 Å). The crystal data, data collection, and structure refinement details of the three polymers are summarized in Table 1. The structures were solved using direct methods and refined using the SHELXL crystallographic software package [22,23]. Final refinements were carried out by the full matrix least-squares methods with anisotropic thermal parameters for nonhydrogen atoms on *F*^2^, while the hydrogen atom positions were located in calculated positions. The hydroxyl H atoms in Polymer 3 were placed in calculated positions and refined as riding, with the O−H distances fixed at 0.82 Å [24]. Selected bonds and angles for Polymers 1–3 are presented in Table 2. The hydrogen-bonding interactions of Polymers 1–3 are listed in Table S1 in the supporting information.

## 3. Results and Discussion

### 3.1. Description of the Structures

#### 3.1.1. Structural Description of [Zn(1,3-BDC)(HL)]_n_ (Polymer 1)

**Figure 3:**
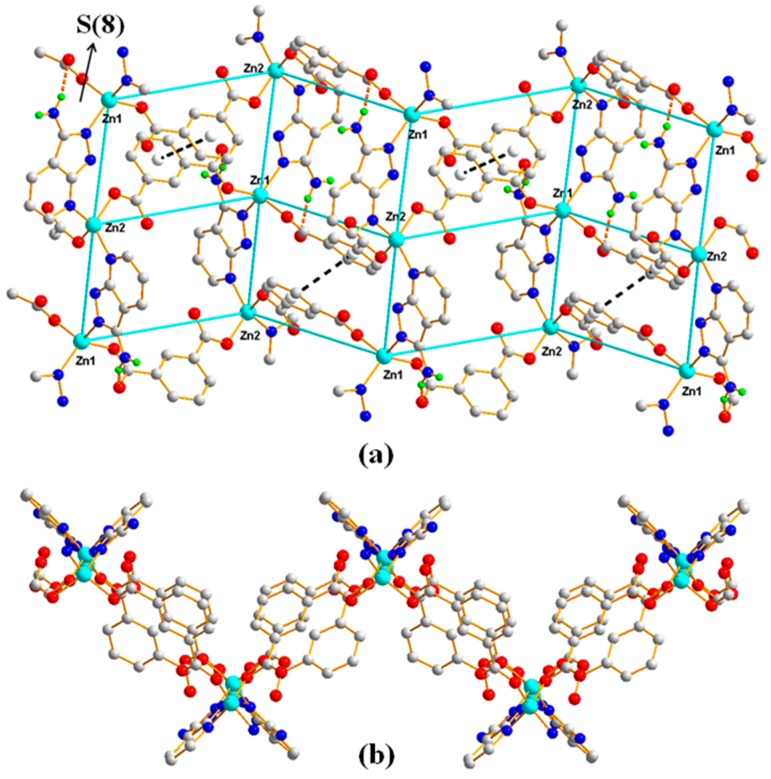
(**a**) The 2D structure of Polymer 1 with the 4-connected sql net highlighted. The dotted lines depict the noncovalent interactions (brown: hydrogen-bonding interactions; black: π–π-stacking interactions) and (**b**) the view down the *a*-axis of the 2D structure.

**Figure 4:**
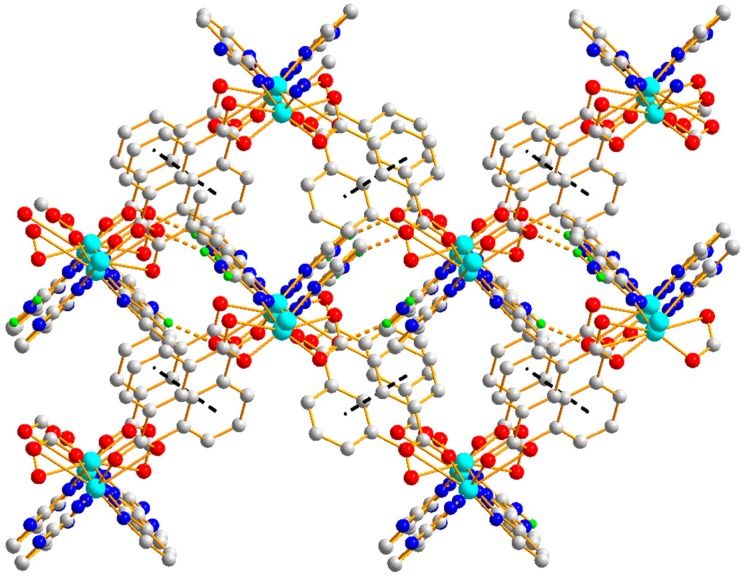
The 3D supramolecular network of Polymer 1 viewed down the *a*-axis.

The single crystal X-ray diffraction analysis revealed that Polymer 1 crystallized in the monoclinic space group *P*2/c. The asymmetric unit contains two Zn(II) ions (Zn1 and Zn2), one 1,3-BDC^2−^ ligand, and one HL ligand. As shown in Figure 2, the Zn1(II) ion is four-coordinated with distorted tetrahedron geometry, which is completed by two carboxylic oxygen atoms from two different 1,3-BDC^2−^ ligands and two pyrazole-N atoms from two different HL ligands (Zn1–O = 1.9572(15) Å and Zn1–N = 2.0421(17) Å). The Zn2 atom is coordinated by two carboxylic oxygen atoms from two different 1,3-BDC^2−^ ligands and two pyridine-N atoms from two different HL ligands, also giving a distorted tetrahedron geometry (Zn2–O = 1.9445(15) Å and Zn2–N = 2.0496(17) Å). In Polymer 1, each 1,3-BDC^2−^ ligand adopts the same coordination fashion—bis-monodentate. The two carboxylate groups in the 1,3-BDC^2−^ ligands act as a monodentate-bridging ligand interlinking the Zn1(II) and Zn2(II) ions alternately into a long-wave chain along the *a*-axis. The neighboring chains are connected by the HL ligands into an ABAB sheet grid-type structure, as shown in Figure 3a (A with two HL ligands in the grid and B with two 1,3-BDC^2−^ ligands in the grid)—S(8) intramolecular hydrogen-bond rings and the π–π-stacking interactions of the aromatic rings further stabilized the grid. Taking each Zn(II) ion as a node, the topology of this structure can be simplified into a familiar 2D 4-connected sql net [25]. The edge lengths of the Zn···Zn separation within the {Zn_4_(HL)_2_(1,3-BDC)_2_} rectangular grid are 6.12 and 9.56 Å and the diagonal Zn···Zn separations are 11.33 and 11.37 Å for the A type grid and 10.57 and 12.08 Å for the B type grid. Viewed down the *a*-axis, the 2D sheet structure presents a zigzag chain along the *c*-axis, as shown in Figure 3b. Two neighboring 2D sheets connected together through N–H···O hydrogen bonds (H(4B)···O(3) = 2.05 Å, N(4)amino···O(3) = 2.86 Å, and < N(4)−H(4B)···O(3) = 158°) generated a 3D supramolecular structure of Polymer 1, as shown in Figure 4.

#### 3.1.2. Structural Description of [Zn_3_(1,3,5-BTC)_2_(HL)_2_(H_2_O)_2_]_n_ (Polymer 2)

**Figure 5:**
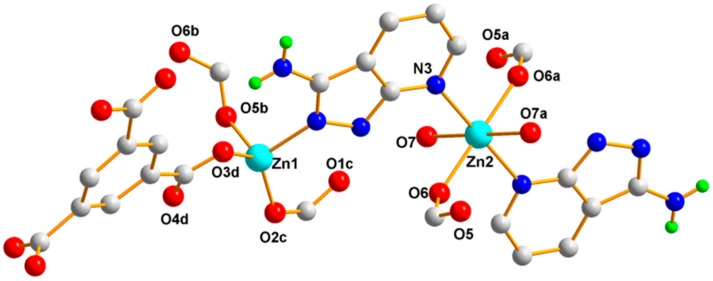
The coordination environment of the Zn(II) ions in Polymer 2. The hydrogen atoms have been omitted for clarity.

**Figure 6:**
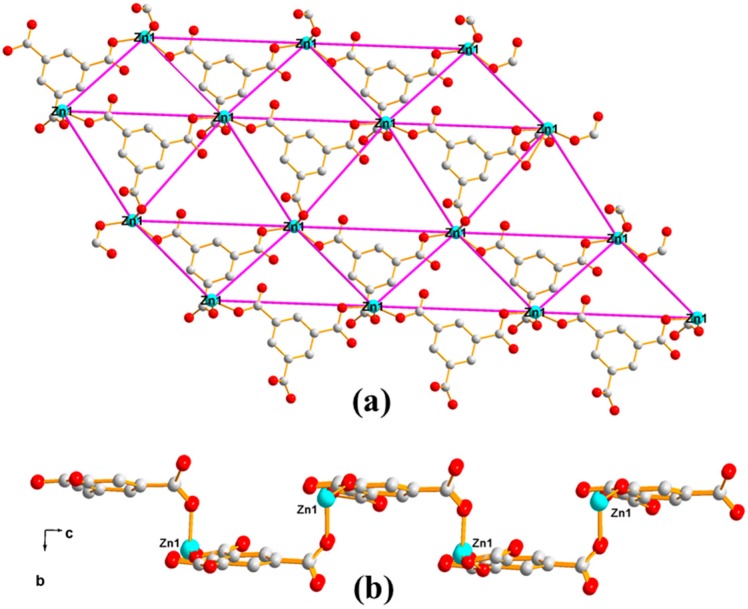
(**a**) Zn1(II) ions connected by the tridentate BTC^3−^ ligands, which form a 2D structure of Polymer 2 viewed down the *b*-axis with the 6-connected net highlighted. (**b**) View down the *a*-axis of the 2D structure showing a square-wave chain structure, which is highlighted by the red line.

**Figure 7:**
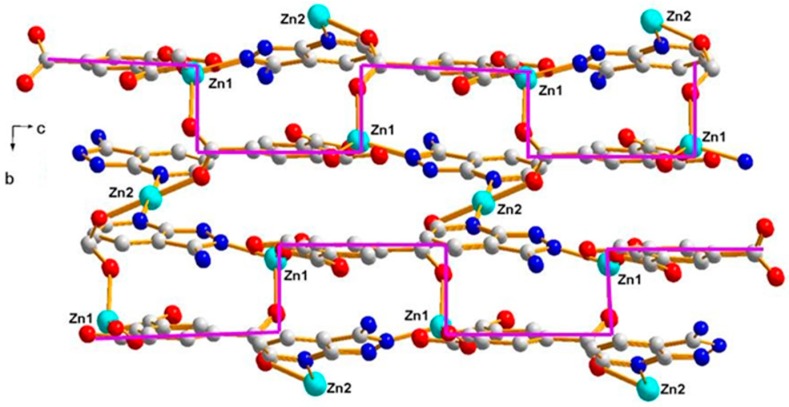
The 3D framework of Polymer 2.

**Figure 8:**
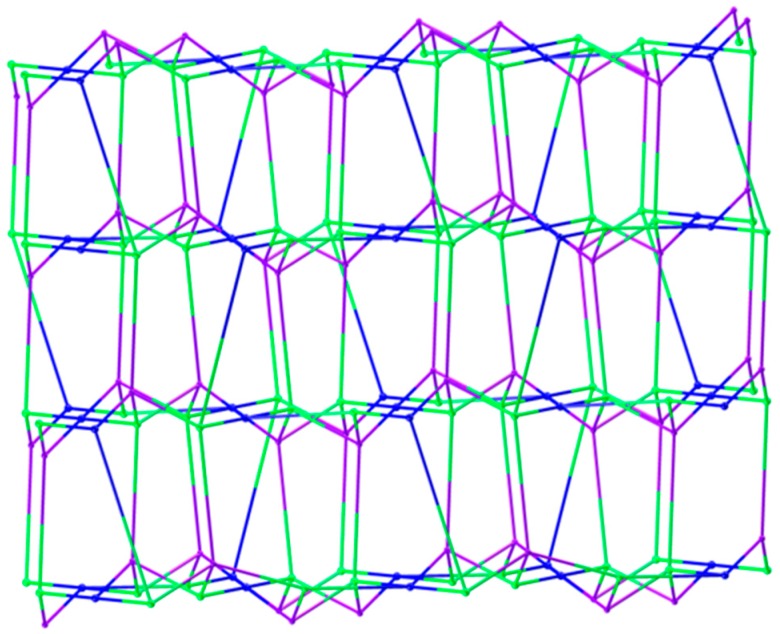
The 3D topological net of Polymer 2 (the blue, green, and purple spheres represent the nodes of Zn2(II), Zn1(II), and BTC^3−^, respectively).

**Figure 9:**
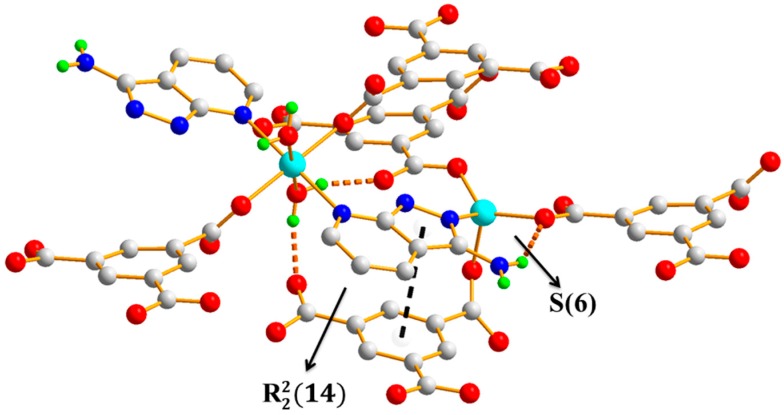
The hydogen bonding rings formed in Polymer 2.

Polymer 2 crystallizes in the monoclinic space group *P*2_1_/c. The asymmetric unit contains two Zn(II) ions (Zn1 and Zn2), one BTC^3−^ ligand, one HL ligand, and one coordination water molecule. As shown in Figure 5, the Zn1(II) ion is four-coordinated with a slightly distorted tetrahedron geometry, which is completed by three carboxylic oxygen atoms from three different BTC^3−^ ligands and one pyrazole-N atom from a HL ligand (Zn1–O = 1.9239(13) − 2.0075(15) Å and Zn1–N = 2.0330(16) Å). The Zn2 atom is coordinated by two carboxylic oxygen atoms from two different BTC^3−^ ligands, two oxygen atoms from two coordination water molecules, and two pyridine-N atoms from two different HL ligands, giving an octahedral geometry with slight distortion (Zn2–O = 2.0596(13) − 2.1648(15) Å and Zn2–N = 2.1997(16) Å). In Polymer 2, each BTC^3−^ ligand adopted the same coordination fashion. There are two kinds of coordination mode of the three carboxylate groups in the BTC^3−^ ligand—two monodentate and one *μ-1,2* [bis(monodentate)] (*syn*–*anti*). The Zn1 atoms are bridged by the tridentate BTC^3−^ ligand to give rise to an undulating 2D 6-connected sheet structure extending in the *a*–*c* plane. Viewed down the *a*-axis, the 2D network shows a square-wave chain structure, as depicted in Figure 6. Crystallographic symmetry of the HL ligands located in two neighboring square-wave-shaped chains links the Zn1(II) and Zn2(II) ions together by the pyrazole-N and pyridine-N atoms, respectively, and generates the 3D framework of Polymer 2. Two symmetrically related *trans* bridging bidentate carboxylic groups connect the Zn1(II) to Zn2(II) ions together, further strengthening the links between two neighboring layers, as shown in Figure 7. From the viewpoint of topology, if the Zn1(II), Zn2(II), and BTC^3−^ are considered as nodes, separately, this structure can be simplified into a 3D (4, 4, 4)-connected net with a Schläfli symbol of (4.5.6^3^.7)2 (5^2^.6^2^.8.9) (5^2^.6^4^), as depicted in Figure 8. Furthermore, intramolecular $R_{2}^{2}$(14) hydrogen-bonding interaction rings are found between the coordination water molecules and the carboxylic oxygen atoms. The N-amino atom from the HL ligands and the carboxylic oxygen atoms also form intramolecular S(6) hydrogen-bonding rings. The π–π interactions are found between the pyrazole ring of the HL ligands and the aromatic ring of the BTC^3−^ ligands with a centroid-to-centroid distance of 3.50 Å, further enhancing the stability of Polymer 2 (Figure 9).

#### 3.1.3. Structural Description of [Zn_9_(5-SO_3_-1,3-BDC)_2_(L)_8_(OH)_4_]_n_ (Polymer 3)

**Figure 10:**
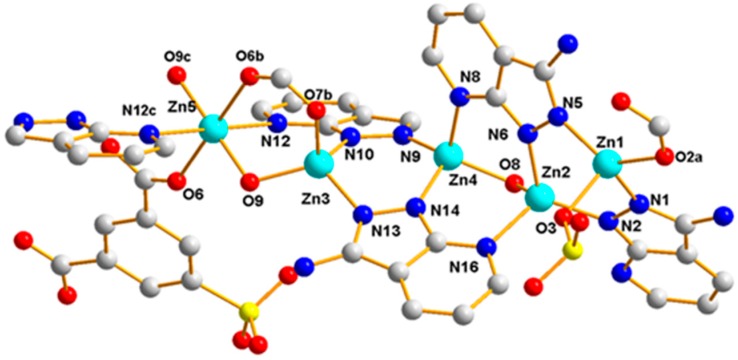
The coordination environment of the Zn(II) ions in Polymer 3. The hydrogen atoms have been omitted for clarity.

**Figure 11:**
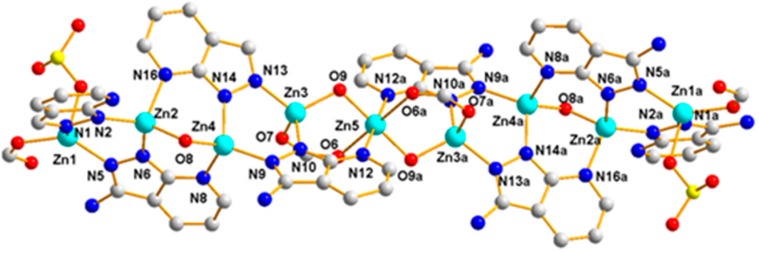
The {Zn_9_(L)_8_(μ_2_-O)_3_(OCO)_2_} unit of Polymer 3.

**Figure 12:**
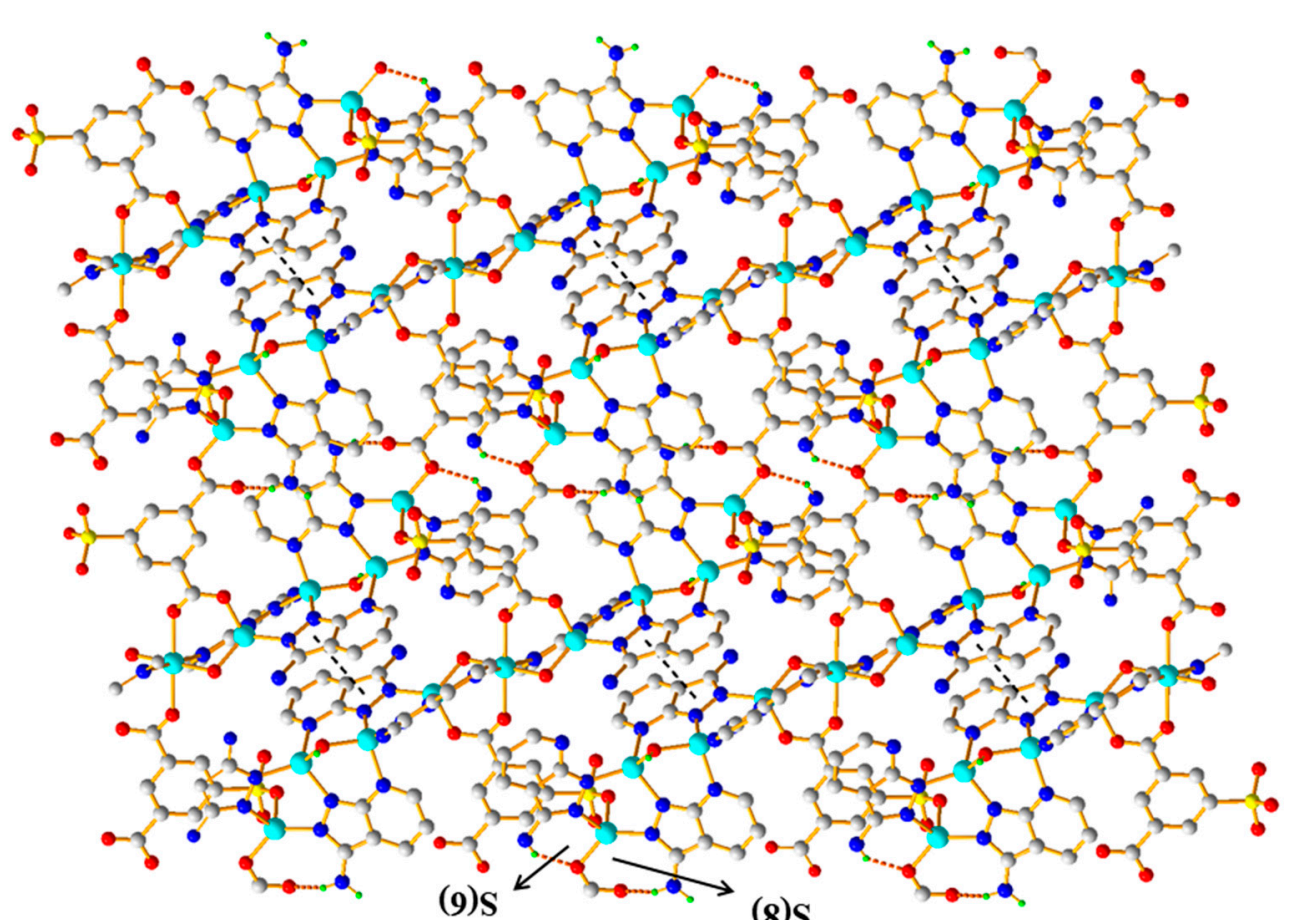
The 2D packing diagram of Polymer 3 constructed by the {Zn_9_} units. The brown dotted lines depict the hydrogen-bonding interactions.

**Figure 13:**
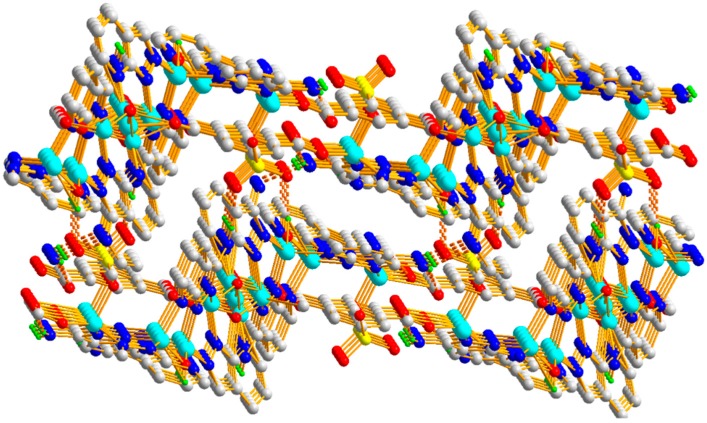
The 3D supramolecular network of Polymer 3.

Polymer 3 crystallizes in the triclinic space group *P*$\bar{1}$. The asymmetric unit contains five Zn(II) ions (Zn1, Zn2, Zn3, Zn4, and Zn5), one 5-SO_3_-1,3-BDC^3−^ ligand, three L^−^ ligands, and two hydroxyl groups. As shown in Figure 10, Zn1(II), Zn2(II), Zn3(II), and Zn4(II) ions are all four-coordinated with slightly distorted tetrahedron geometries: the Zn1(II) ion is completed by one carboxylic oxygen atom from the 5-SO_3_-BDC^3−^ ligand, one oxygen atom from the sulfo group of the 5-SO_3_-BDC^3−^ ligand, and two pyrazole-N atoms from the L^−^ ligand (Zn1–O = 1.934(3) – 1.997(4) Å and Zn1–N = 1.991(4) – 2.006(4) Å); the Zn2(II) ion is coordinated by one *μ*_2_-hydroxyl oxygen atom, two pyrazole-N atoms, and one pyridine-N atom from three different HL ligands; the Zn3(II) ion is coordinated by one carboxylic oxygen atom from the 5-SO_3_-BDC^3−^ ligand, one *μ*_2_-hydroxyl oxygen atom, and two pyrazole-N atoms from two different HL ligands; and the Zn4(II) ion is coordinated by one *μ*_2_-hydroxyl oxygen atom, two pyrazole-N atoms, and one pyridine-N atom from three different L^−^ ligands. The Zn5(II) ions present a six-coordinated mode surrounded by two carboxylic oxygen atoms from two different 5-SO_3_-BDC^3−^ ligands, two *μ*_2_-hydroxyl oxygen atoms, and two pyridine-Ns, giving an octahedral geometry with considerable distortion. The distances of the Zn–O/N and angles of Zn–O/N–Zn are presented in Table 2. Zn1(II), Zn2(II), Zn4(II), and Zn3(II) ions and their related symmetry, Zn1a(II), Zn2a(II), Zn4a(II), Zn3a(II), are distributed on the two sides of the Zn5(II) ion. These nine metal ions are arranged in a quasi-linear form of Zn1-Zn2-Zn4-Zn3-Zn5-Zn3a-Zn4a-Zn2a-Zn1a (*a* = 2 − *x*, 1 − *y*, −*z*), forming a {Zn_9_(L)_8_(*μ*_2_-OH)_4_(OCO)_2}_ secondary building unit ({Zn_9_} unit), as shown in Figure 11. Although some nonanuclear polymers have been reported [26,27,28,29], most of them are based on rare earth ions [27,30,31,32], while those based on the Zn(II) ion are relatively rare [33,34]. Furthermore, most of the reported structures of nonanuclear units are a square antiprismatic arrangement or hourglass structure [33,35]. As far as we know, those based on the quasi-linear array of a nonanuclear zinc polymer have not been reported. The adjacent {Zn_9_(L)_8_(μ_2_-OH)_4_(OCO)_2_} units are connected to a two-dimensional planar structure by the 5-SO_3_-BDC^3−^ ligand. Intramolecular hydrogen-bonding S(6) and S(8) rings as well as π–π interactions between the neighboring pyrazole–pyrazole rings of two L^−^ ligands with a centroid-to-centroid distance of 3.72 Å stabilized the 2D sheet of Polymer 3, as depicted in Figure 12. The 2D sheet was further extended by intermolecular N–H···N (H(7B)···N(12) amino = 2.39 Å, N(7)amino···N(12) amino = 3.19 Å, and < N(7) amino−H(7B)···N(12) amino = 156°) and O–H···O (H(8)···O(4) sulfo = 1.98 Å, O(8) hydroxyl···O(4) sulfo = 2.83 Å, and < O(8) hydroxyl−H(8)···O(4) sulfo = 175°) hydrogen-bonding interactions, resulting in a 3D supramolecular structure of Polymer 3. The π–π interactions between the neighboring pyridine−pyridine rings of two L^−^ ligands with a centroid-to-centroid distance of 3.51 Å further stabilized the 3D supramolecular structure of Polymer 3 (Figure 13).

#### 3.1.4. Comparison of the Structures of Polymers 1–3

As mentioned above, it can be seen that covalent interactions, hydrogen-bonding interactions, as well as π···π-stacking interactions play important roles in constructing the structures of supramolecular coordination polymers. The 5-positioned substituted functional group of 1,3-benzenedicarboxylic acid plays a significant role in determining the resulting structures. The different coordination modes of the HL ligand allow it to play different roles in constructing the three polymers. In Polymers 1 and 2, the pyridine-N atom and one of the pyrazole-N atoms of the HL ligand are involved in coordination and play a pivotal role in increasing the dimensionality of Polymers 1 and 2. In Polymer 3, in order to balance the positive charge of the metal ions, each HL ligand in Polymer 3 is deprotonated. The pyridine-N atom and two pyrazole-N atoms in each L^−^ ligand are all coordinated with Zn(II) centers and make the novel {Zn_9_} units of Polymer 3.

### 3.2. PXRD and Photoluminescence Properties of the Three Polymers

The phase purity of the three polymers was detected by using powder X-ray diffraction (PXRD) experiments. The peaks of the experimental and simulated PXRD patterns coincide with each other, which indicate the purity of the three polymers (Figures S1–S3).

**Figure 14:**
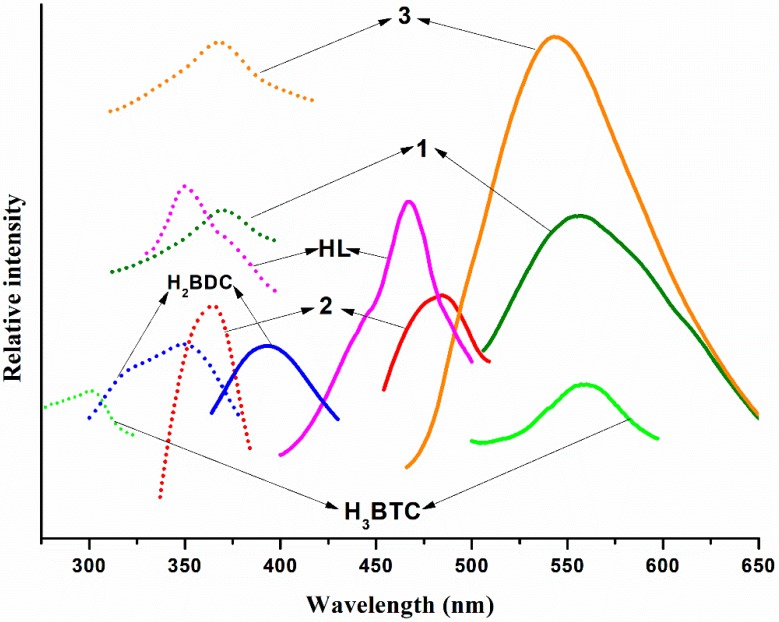
Excitation and emission spectra of the three polymers—dot symbol (EX) and solid symbol (EM).

The d^10^ coordination polymers were proved to have good photoluminescence properties. Therefore, the solid-state photoluminescence properties of Polymers 1–3, as well as the free ligand, were studied at ambient temperature. As shown in Figure 14, when the excitation spectrum was excited at 350 nm, the free HL gave an optical emission bond at 467 nm. The photoluminescent spectra of the free H_2_BDC, H_3_BTC, and 5-SO_3_Na-H_2_BDC ligands were also investigated—strong emission bands were observed at 393 nm and 560 nm for the free H_2_BDC and H_3_BTC ligands, respectively (λex = 349 nm for the H_2_BDC ligand; λex = 301 nm for the free H_3_BTC ligand). No luminescence spectrum was observed for the free 5-SO_3_Na-H_2_BDC ligands—this phenomenon may be due to the presence of the SO_3_Na group. The optimal emission bands were detected at 558 nm for Polymer 1, 485 nm for Polymer 2, and 543 nm for Polymer 3 (*λ*_ex_ = 373 nm for Polymer 1, *λ*_ex_ = 368 nm for Polymer 2, and *λ*_ex_ = 370 nm for Polymer 3). The emission peaks of Polymers 1–3 were similar to that of the HL ligand, therefore, their emissions may be ascribed to the ligand-centered π–π* transitions of the HL ligand [36].

### 3.3. Thermogravimetric Analyses (TGA) of the Three Polymers

To investigate the thermal stabilities of Polymers 1–3, thermogravimetric analyses were performed in N_2_ atmosphere. As shown in Figure S4 in the supporting information, Polymer 1 was stable up to 420 °C. The loss of 4.02% (calcd 3.94%) in 185–341 °C of Polymer 2 corresponds to the removal of coordination water molecules. The loss of 3.04% (calcd 3.08%) in 185–341 °C of Polymer 3 corresponds to the removal of hydroxyl in the molecules. Their further obvious weight losses were ascribed to the collapse of their frameworks.

## 4. Conclusions

In conclusion, three new supramolecular coordination polymers with different architectures were successfully synthesized by assembling multidentate HL ligand and Zn^2+^ ions in the presence of different aromatic polycarboxylate ligands. Although the three polymers were synthesized under similar conditions, their structures were quite different, thus it can be seen that the 5-substituted functional group of 1,3-benzenedicarboxylate had a great influence on the final structures. Even so, the influence of noncovalent bonds (hydrogen-bonding interactions and π···π -stacking interactions) on the structure of coordination polymers should not be underestimated. This work provides a promising approach on the construction of novel supramolecular coordination polymers with multidentate N-containing ligands and aromatic polycarboxylates.

## Figures and Tables

**Table 1:** Crystal data and structure refinement parameters for the three polymers.

Polymer	1	2	3
Empirical formula	C_14_H_10_N_4_O_4_Zn	C_30_H_22_N_8_O_14_Zn_3_	C_64_H_50_N_32_O_18_S_2_Zn_9_
Formula weight	363.63	914.67	2207.81
Temp (K)	293(2)	293(2)	293(2)
Crystal system	Monoclinic	Monoclinic	Triclinic
Space group	*P*2/c	*P*2_1_/c	*P*$\bar{1}$
*a* (Å)	12.1658(3)	9.7940(8)	9.9428(7)
*b* (Å)	8.5041(3)	13.4881(11)	13.4612(8)
*c* (Å)	18.0701(4)	14.9135(9)	15.1197(10)
*α* (°)	90	90	73.567(2)
*β* (°)	126.7210(10)	126.970(4)	84.738(3)
*γ* (°)	90	90	74.256(2)
V (Å^3^)	3878.9(2)	1574.0(2)	1867.9(2)
*Z*	4	2	1
*F*(000)	736	920	1104
Density (Mg/m^3^)	1.612	1.930	1.963
Absorption Coefficients (mm^−1^)	1.664	2.357	1.093
data/restraints/params	3443/0/209	3617/0/250	6416/0/565
GOF	0.999	1.035	1.064
*R*_1_ [I > 2*σ*(I)]	0.0294	0.0242	0.0522
*wR*_2_ [I > 2*σ*(I)]	0.0946	0.0629	0.1026

^a^*R*_1_ = ∑(||*F*_o_ − *F*_c_||)/∑|*F*_o_| and *wR*_2_ = [∑*w*(*F*_o_^2^ − *F*_c_^2^)^2^/∑*w*(*F*_o_^2^)^2^]^1/2^.

**Table 2:** Selected bonds (Å) and angles (°) for Polymers 1–3.

**Polymer 1**			
Zn(1)-O(2)#1	1.9572(15)	Zn(2)-O(4)	1.9445(15)
Zn(1)-O(2)	1.9572(15)	Zn(2)-N(3)#3	2.0496(17)
Zn(1)-N(1)	2.0421(17)	Zn(2)-N(3)#4	2.0496(17)
Zn(1)-N(1)#1	2.0421(17)	Zn(2)-O(4)#2	1.9445(15)
N(3)-Zn(2)#3	2.0496(17)		
O(4)#2-Zn(2)-O(4)	103.18(10)	O(2)#1-Zn(1)-O(2)	107.29(10)
O(4)#2-Zn(2)-N(3)#3	100.44(6)	O(2)#1-Zn(1)-N(1)	117.82(7)
O(4)-Zn(2)-N(3)#3	124.75(7)	O(2)-Zn(1)-N(1)	102.43(7)
O(4)#2-Zn(2)-N(3)#4	124.75(7)	O(2)#1-Zn(1)-N(1)#1	102.43(7)
O(4)-Zn(2)-N(3)#4	100.44(6)	O(2)-Zn(1)-N(1)#1	117.82(7)
N(3)#3-Zn(2)-N(3)#4	105.60(10)	N(1)-Zn(1)-N(1)#1	109.77(11)
**Polymer 2**			
Zn(1)-O(2)	1.9239(13)	Zn(1)-O(3)#1	1.9713(15)
Zn(1)-O(5)#2	2.0075(15)	Zn(1)-N(1)	2.0330(16)
Zn(2)-O(6)	2.0596(13)	Zn(2)-O(6)	2.0596(13)
Zn(2)-O(7)#3	2.1648(15)	Zn(2)-O(7)	2.1648(15)
Zn(2)-N(3)#4	2.1997(16)	Zn(2)-N(3)#5	2.1997(16)
O(3)-Zn(1)#6	1.9713(15)	O(5)-Zn(1)#7	2.0075(15)
O(2)-Zn(1)-O(3)#1	131.93(7)	O(2)-Zn(1)-O(5)#2	100.29(6)
O(3)#1-Zn(1)-O(5)#2	107.37(7)	O(2)-Zn(1)-N(1)	114.47(6)
O(3)#1-Zn(1)-N(1)	93.61(6)	O(5)#2-Zn(1)-N(1)	107.79(6)
O(6)-Zn(2)-O(6)#3	180.000(1)	O(6)-Zn(2)-O(7)#3	99.04(6)
O(6)#3-Zn(2)-O(7)#3	80.96(6)	O(6)-Zn(2)-O(7)	80.96(6)
O(6)#3-Zn(2)-O(7)	99.04(6)	O(7)#3-Zn(2)-O(7)	180.000(1)
O(6)-Zn(2)-N(3)#4	92.84(6)	O(6)#3-Zn(2)-N(3)#4	87.16(6)
O(7)#3-Zn(2)-N(3)#4	86.50(6)	O(7)-Zn(2)-N(3)#4	93.50(6)
O(6)-Zn(2)-N(3)#5	87.16(6)	O(6)#3-Zn(2)-N(3)#5	92.84(6)
O(7)#3-Zn(2)-N(3)#5	93.50(6)	O(7)-Zn(2)-N(3)#5	86.50(6)
N(3)#4-Zn(2)-N(3)#5	180.00(8)		
**Polymer 3**			
Zn(1)-O(2)#1	1.934(3)	Zn(3)-N(13)	1.965(4)
Zn(1)-N(5)	1.991(4)	Zn(3)-O(7)#2	1.974(4)
Zn(1)-O(3)	1.997(4)	Zn(3)-N(10)	1.976(5)
Zn(1)-N(1)	2.006(4)	Zn(3)-Zn(5B)#3	3.045(17)
Zn(2)-O(8)	1.922(4)	Zn(3)-Zn(5)	3.1242(6)
Zn(2)-N(2)	1.949(4)	Zn(4)-O(8)	1.911(4)
Zn(2)-N(6)	2.023(4)	Zn(4)-N(9)	1.978(4)
Zn(2)-N(16)	2.056(5)	Zn(4)-N(14)	1.990(5)
Zn(2)-Zn(4)	3.0695(9)	Zn(4)-N(8)	2.053(4)
Zn(3)-O(9)	1.909(4)	Zn(5)-O(9)#4	1.899(4)
Zn(5)-O(9)	1.899(4)	Zn(5B)-O(9)#6	1.998(16)
Zn(5)-O(6)#3	2.183(4)	Zn(5B)-O(9)#3	2.121(17)
Zn(5)-O(6)#2	2.183(4)	Zn(5B)-O(6)	2.170(18)
Zn(5)-Zn(3)#4	3.1242(6)	Zn(5B)-O(6)#5	2.469(16)
Zn(5B)-Zn(5B)#5	1.597(14)	Zn(5B)-Zn(3)#3	3.045(17)
Zn(5B)-N(12)#3	1.743(9)	O(7)-Zn(3)#6	1.974(4)
O(6)-Zn(5B)#5	2.469(16)	O(6)-Zn(5)#6	2.183(4)
O(2)#1-Zn(1)-N(5)	133.06(17)	O(3)-Zn(1)-N(1)	96.91(17)
O(2)#1-Zn(1)-O(3)	107.56(16)	O(8)-Zn(2)-N(2)	122.63(17)
N(5)-Zn(1)-O(3)	103.45(16)	O(8)-Zn(2)-N(6)	102.80(18)
O(2)#1-Zn(1)-N(1)	100.10(16)	N(2)-Zn(2)-N(6)	110.20(18)
N(5)-Zn(1)-N(1)	110.24(17)	O(8)-Zn(2)-N(16)	100.61(17)
N(6)-Zn(2)-N(16)	107.41(19)	N(2)-Zn(2)-N(16)	111.89(19)
O(8)-Zn(2)-Zn(4)	36.67(11)	O(9)-Zn(3)-N(13)	116.15(18)
N(2)-Zn(2)-Zn(4)	158.92(14)	O(9)-Zn(3)-O(7)#2	104.77(17)
N(6)-Zn(2)-Zn(4)	78.68(12)	N(13)-Zn(3)-O(7)#2	105.91(17)
N(16)-Zn(2)-Zn(4)	81.99(12)	O(9)-Zn(3)-N(10)	112.25(19)
O(7)#2-Zn(3)-N(10)	107.68(19)	N(13)-Zn(3)-N(10)	109.45(19)
O(9)-Zn(3)-Zn(5B)#3	43.6(3)	Zn(5B)#3-Zn(3)-Zn(5)	14.80(13)
N(10)-Zn(3)-Zn(5B)#3	75.1(2)	O(8)-Zn(4)-N(9)	125.13(19)
O(9)-Zn(3)-Zn(5)	34.78(12)	O(8)-Zn(4)-N(14)	101.16(18)
N(13)-Zn(3)-Zn(5)	150.93(13)	N(9)-Zn(4)-N(14)	111.05(19)
O(7)#2-Zn(3)-Zn(5)	88.18(11)	O(8)-Zn(4)-N(8)	104.06(18)
N(10)-Zn(3)-Zn(5)	89.41(13)	N(9)-Zn(4)-N(8)	108.08(18)
N(14)-Zn(4)-N(8)	105.74(19)	O(9)#4-Zn(5)-O(6)#2	88.51(16)
O(8)-Zn(4)-Zn(2)	36.92(12)	O(9)-Zn(5)-O(6)#2	91.49(16)
N(9)-Zn(4)-Zn(2)	162.04(14)	O(6)#3-Zn(5)-O(6)#2	180.0
N(14)-Zn(4)-Zn(2)	78.57(12)	O(9)#4-Zn(5)-Zn(3)#4	34.99(12)
N(8)-Zn(4)-Zn(2)	82.59(12)	O(9)-Zn(5)-Zn(3)#4	145.01(12)
O(9)#4-Zn(5)-O(9)	180.0	O(6)#3-Zn(5)-Zn(3)#4	66.42(11)
O(9)#4-Zn(5)-O(6)#3	91.49(16)	O(6)#2-Zn(5)-Zn(3)#4	113.58(11)
O(9)-Zn(5)-O(6)#3	88.51(16)	O(9)#4-Zn(5)-Zn(3)	145.01(12)
O(9)-Zn(5)-Zn(3)	34.99(12)	N(12)#3-Zn(5B)-O(9)#6	115.2(8)
O(6)#3-Zn(5)-Zn(3)	113.58(11)	Zn(5B)#5-Zn(5B)-O(9)#3	63.2(11)
O(6)#2-Zn(5)-Zn(3)	66.42(11)	N(12)#3-Zn(5B)-O(9)#3	110.0(8)
Zn(3)#4-Zn(5)-Zn(3)	180.0	O(9)#6-Zn(5B)-O(9)#3	134.5(4)
Zn(5B)#5-Zn(5B)-N(12)#3	170.6(18)	Zn(5B)#5-Zn(5B)-O(6)	80.4(12)
Zn(5B)#5-Zn(5B)-O(9)#6	71.3(11)	O(6)-Zn(5B)-O(6)#5	140.4(3)
N(12)#3-Zn(5B)-O(6)	105.9(7)	Zn(5B)#5-Zn(5B)-Zn(3)#3	88.2(12)
O(9)#6-Zn(5B)-O(6)	89.3(6)	N(12)#3-Zn(5B)-Zn(3)#3	82.7(6)
O(9)#3-Zn(5B)-O(6)	83.5(6)	O(9)#6-Zn(5B)-Zn(3)#3	143.9(7)
Zn(5B)#5-Zn(5B)-O(6)#5	60.0(11)	O(9)#3-Zn(5B)-Zn(3)#3	38.4(3)
N(12)#3-Zn(5B)-O(6)#5	113.4(8)	O(6)-Zn(5B)-Zn(3)#3	117.0(7)
O(9)#6-Zn(5B)-O(6)#5	78.7(5)	O(6)#5-Zn(5B)-Zn(3)#3	65.2(4)
O(9)#3-Zn(5B)-O(6)#5	78.9(5)		

Symmetry transformations used to generate equivalent atoms: #1 −*x*, *y*, −*z* + 1/2; #2 −*x* + 1, *y*, −*z* + 3/2; #3 −*x* + 1, −*y*, −*z* + 1; and #4 *x*, −*y*, *z* + 1/2 for Polymer 1; #1 *x* + 1, *y*, *z*; #2 *x*, −*y* + 3/2, *z* − 1/2; #3 −*x* + 2, −*y* + 2, −*z* + 3; #4 −*x* + 1, −*y* + 2, −*z* + 2; #5 *x* + 1, *y*, *z* + 1; #6 *x* − 1, *y*, *z*; #7 *x*, −*y* + 3/2, *z* + 1/2; and #8 *x* − 1, *y*, *z* − 1 for Polymer 2; #1 −*x* + 2, −*y* + 2, −*z*; #2 *x*, *y* − 1, *z*; #3 −*x* + 1, −*y* + 1, −*z* + 1; #4 −*x* + 1, −*y*, −*z* + 1; #5 −*x* + 1, −*y* + 2, −*z* + 1; and #6 *x*, *y* + 1, *z* for Polymer 3.

## Supplementary Materials

The following are available online at https://www.mdpi.com/2073-4360/11/5/819/s1: Figure S1. The simulated and experimental PXRD of Polymer 1; Figure S2. The simulated and experimental PXRD of Polymer 2; Figure S3. The simulated and experimental PXRD of Polymer 3; Figure S4. TGA curves for the three polymers; Table S1. Hydrogen-bond geometry (Å, °) of the three polymers.
