# Supplementary material for: Three New Supramolecular Coordination Polymers Based on 1H-pyrazolo[3,4-b]pyridin-3-amine and 1,3-benzenedicarboxylate Derivatives
Source: Polymers (Basel). 2019 May 7;11(5):819. doi: 10.3390/polym11050819 (PMC6571696; doi:10.3390/polym11050819)
Supplement: Supplementary file 1 [file polymers-11-00819-s001.pdf]

Supporting information

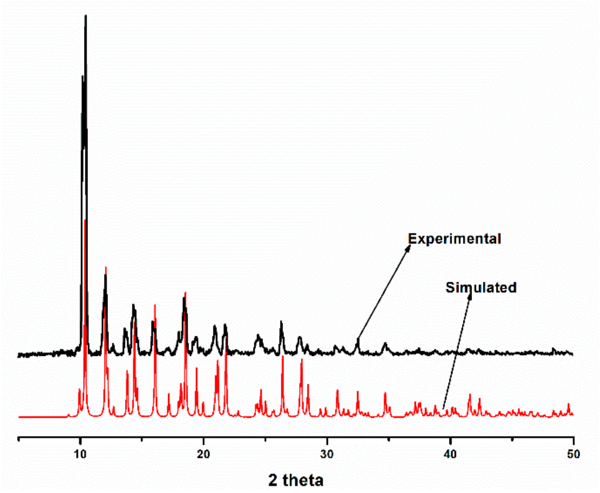

Figure S1**.** The simulated and experimental PXRD of the Polymer 1**.**

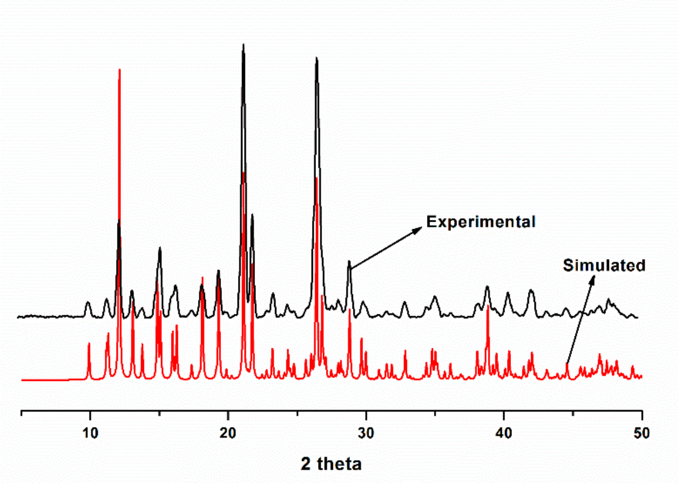

Figure S2. The simulated and experimental PXRD of the Polymer 2**.**

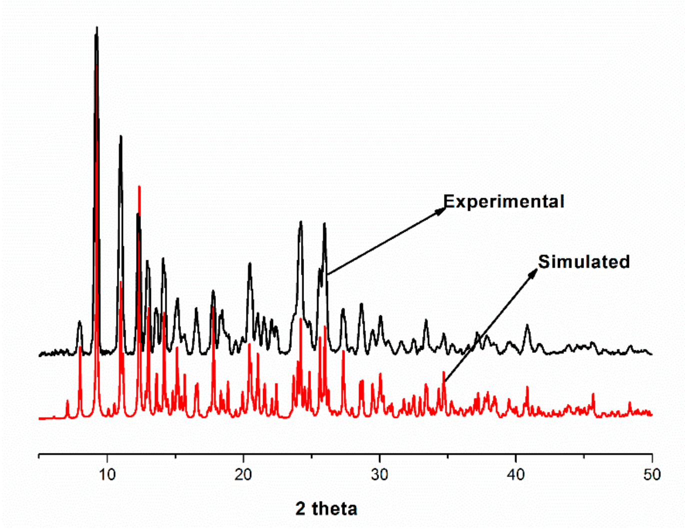

Figure S3. The simulated and experimental PXRD of the Polymer 3.

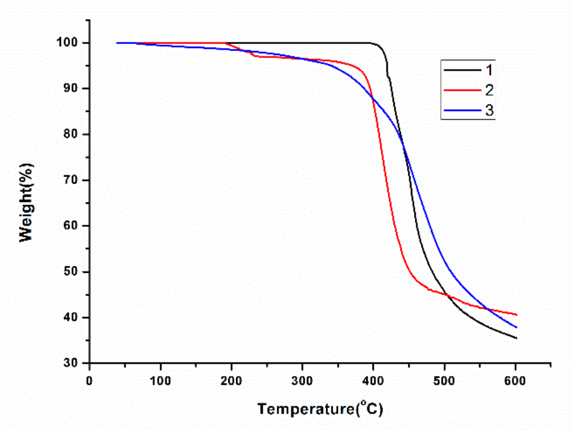

Figure S4. TGA curves for three polymers.

Table S1. Hydrogen-bond geometry (Å, º) of the three polymers.

D—H···*A*	H···*A*	*D*···*A*	*D*—H···*A*
		**1**	
N2—H···O4^{i}	2.01	2.743 (1)	142
N4—H4A···O1	2.17	2.936(5)	147
N4—H4B···O3^{ii}	2.05	2.863(8)	158
		**2**	
N4—H4A···O3i	2.19	2.931(2)	142
O7—H7···O1^{ii}	2.01	2.837(2)	167
O7—H8···O4^{iii}	1.91	2.743(2)	169
		**3**	
N3—H3A···O2^{i}	2.14	2.901(7)	147
N7—H7A···O1^{i}	2.02	2.798(8)	150
N7—H7B···N12^{ii}	2.39	3.193(9)	156
O8—H8···O4^{iii}	1.98	2.826(6)	175
N15—H15B···O5^{iv}	2.14	2.994(8)	176

Symmetry codes: (i) x, −y, z-1/2 (ii) x, 1+y, z for **1**; (i) x+1, y, z, (ii) −x+1, −y+2, −z+2, (iii) x−1, y+1/2,5/2-z for **2**; (i) 2-x,2-y,-z (ii) 1-x, 1-y, -z. (iii) x−1, y, z, (iv) -x+2, -y+1, -z+1 for **3**.
